# The effects of competition on fitness depend on the sex of both competitors

**DOI:** 10.1002/ece3.6620

**Published:** 2020-09-12

**Authors:** Maider Iglesias‐Carrasco, Samuel Brookes, Loeske E. B. Kruuk, Megan L. Head

**Affiliations:** ^1^ Division of Ecology and Evolution Research School of Biology Australian National University Canberra ACT Australia

**Keywords:** *Callosobruchus maculatus*, fitness, intraspecific competition, sex‐specific, social environment

## Abstract

In intraspecific competition, the sex of competing individuals is likely to be important in determining the outcome of competitive interactions and the way exposure to conspecifics during development influences adult fitness traits. Previous studies have explored differences between males and females in their response to intraspecific competition. However, few have tested how the sex of the competitors, or any interactions between focal and competitor sex, influences the nature and intensity of competition. We set up larval seed beetles *Callosobruchus maculatus* to develop either alone or in the presence of a male or female competitor and measured a suite of traits: development time, emergence weight; male ejaculate mass, copulation duration, and lifespan; and female lifetime fecundity, offspring egg–adult survival, and lifespan. We found effects of competition and competitor sex on the development time and emergence weight of both males and females, and also of an interaction between focal and competitor sex: Females emerged lighter when competing with another female, while males did not. There was little effect of larval competition on male and female adult fitness traits, with the exception of the effect of a female competitor on a focal female's offspring survival rate. Our results highlight the importance of directly measuring the effects of competition on fitness traits, rather than distant proxies for fitness, and suggest that competition with the sex with the greater resource requirements (here females) might play a role in driving trait evolution. We also found that male–male competition during development resulted in shorter copulation times than male–female competition, a result that remained when controlling for the weight of competitors. Although it is difficult to definitively tease apart the effects of social environment and access to resources, this result suggests that something about the sex of competitors other than their size is driving this pattern.

## Graphical abstract

Using the seed beetle *Callosobruchus maculatus* as a model, we explore whether the sex of the focal individual, the sex of the competitor, and the interaction between the focal and the competitor sex explain the way competition affects the development of adult fitness traits. We conclude that that competition with the sex with higher competitive ability might have strong effects in driving the expression of traits.

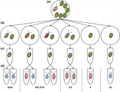

## 1 INTRODUCTION

Early environmental conditions can change the developmental trajectories of juveniles and hence can have significant effects on adult trait expression and fitness (Byrne et al., 2009; Fischer, Bot, Brakefield, & Zwaan, 2003; Mayntz, Toft, & Vollrath, 2003; Relyea, 2004). In particular, competition with conspecifics (i.e., intraspecific competition), which occurs when individuals compete for limited resources, might be particularly important for the development of life‐history and morphological traits (Han & Brooks, 2015; Stockley & Seal, 2001; Vamosi, 2005). Since intraspecific competition can create winners and losers, it is likely to lead to between‐individual variation in the expression of resource‐dependent traits, behaviors, and, importantly, fitness.

An important characteristic of individuals that influences their resource requirements for survival and reproduction, as well as their impact on the ability of other individuals to acquire the resources *they* need, is their biological sex. Males and females differ in many morphological, physiological, and behavioral traits that have the potential to influence both their requirements and their impact on conspecifics, and hence the outcome of competitive interactions (Bedhomme, Agnew, Sidobre, & Michalakis, 2003; Nicolaus et al., 2009; Varga & Kytöviita, 2012). Such differences between males and females could lead to one sex suffering more from competition than the other. For example, in unfavorable conditions (e.g., limited food resources), the smaller sex is often disadvantaged due to the larger sex gaining resources at their expense (Hipkiss, Hörnfeldt, Eklund, & Berlin, 2002; Oddie, 2000; Råberg, Stjernman, & Nilsson, 2005; Rowland, Love, Verspoor, Sheldon, & Williams, 2007). However, in other cases, the larger sex may be disadvantaged, due to the higher energy requirements of being large (Bonneaud et al., 2016; Laaksonen et al., 2004; Wikelski & Thom, 2000). Although a few studies consider the effect of competitor sex on competitive interactions (Bonisoli‐Alquati, Boncoraglio, Caprioli, & Saino, 2011; Brookes, Iglesias‐Carrasco, Kruuk, & Head, In press), studies typically consider the sex‐specific responses of focal individuals and ignore the role of competitor sex (Nicolaus et al., 2009; Oddie, 2000). This distinction between the sex of focal and competitor individuals is important because by ignoring competitor sex, these studies also ignore the potential for one sex to affect the other sex differently.

To understand how differences between males and females shape evolutionary responses to competition, it is necessary to explore how the sex of focal and competitor individuals interacts to influence an individual's total lifetime fitness or at least life‐history traits that are closely correlated with lifetime fitness—hereafter “fitness traits.” To date, most studies have focused on how competition affects the development of morphological traits (e.g., foraging structures, Relyea & Auld, 2005; body shape, Van Buskirk, 2009; leaf structure, Bennett, Riibak, Tamme, Lewis, & Pärtel, 2016), and often ignore the long‐term effects on fitness (but see, e.g., Relyea & Hoverman, 2003; Steets, Salla, & Ashman, 2017). Although there may be costs associated with social dominance (Bell, Nichols, Gilchrist, Cant, & Hodge, 2012), selection should favor competitive individuals that gain more resources to invest in reproduction and hence that have higher fitness than less competitive individuals. While it may be difficult to measure fitness components in many study species, this lack of studies addressing the fitness consequences of competition is unfortunate, because ultimately the outcome of competition will determine which individuals contribute genes to future generations and hence determine how phenotypes evolve in response to competition.

In addition to the effects that direct competition has on individuals through alteration of access to food and resources, the presence of other individuals during development may also influence the sex‐specific expression of fitness traits through perceived differences in the social environment. For example, the presence of other males can alter male developmental trajectories and affect their investment in reproductive traits (e.g., sperm) due to variation in the perceived strength of future reproductive competition (reviewed in Bretman, Gage, & Chapman, 2011). Although effects of juvenile social environments on female adult traits tend to be less well studied than for males (Bailey, Gray, & Zuk, 2010; DiRienzo, Pruitt, & Hedrick, 2012; Gray & Simmons, 2013, but see Kasumovic, Hall, Try, & Brooks, 2011), females have also been shown to alter investment in reproduction in response to the perceived strength of reproductive competition. For example, female common gobies (*Pomatoschistus microps*) increase clutch sizes in response to predicted limited access to males (Heubel, Lindström, & Kokko, 2008). As such, the effect that the sex of individuals plays in intraspecific competition and its fitness consequences is likely to be complex due to the interaction between competition for resources and the strategic allocation of resources in response to the social environment.

The seed beetle, *Callosobruchus maculatus*, is an ideal model species with which to compare the fitness consequences of the presence of conspecifics during development for males and females. Females oviposit on the surface of beans, and when eggs hatch, the larvae burrow into the bean, where they then feed, develop, and ultimately pupate (Stillwell & Fox, 2007). Adults do not need to eat or drink, and all food resources required for development are acquired during the time within the bean (Stillwell & Fox, 2007). This means that the entirety of intraspecific competition for food resources occurs during the larval stages. “Sharing” a bean, that is, when more than one larva is developing within a single bean, often leads to a reduction in emergence weight, especially in females (Iglesias‐Carrasco, Jennions, Zajitschek, & Head, 2018). This difference between the sexes might be a consequence of strong sexual dimorphism in seed beetles: Females are larger than males (Colgoni & Vamosi, 2006; Rankin & Arnqvist, 2008) and take longer to develop (Hallsson & Björklund, 2012). While being large might be advantageous for females due to greater fertility (Chou, Iwasa, & Nakazawa, 2016), males might benefit from having a smaller body size because it allows them to emerge early, and hence, to have access to virgin females (Hallsson & Björklund, 2012). This large sexual size dimorphism and variation in biological requirements between males and females, in combination with a short adult lifespan, thus make *C. maculatus* suitable to study the interactive effects of the sex of the competitor and focal individual on fitness.

Here, we test for sex‐specific responses to the presence of a conspecific in *C. maculatus* by raising individuals of both sexes either alone (one larva per bean) or in the presence of a competitor of the same or opposite sex (two larvae per bean). We predict that (a) the presence of a competitor will negatively impact fitness traits, independently of the sex of either the focal or competitor individual. Since females are larger than males, we also predict that (b) females will show greater plastic responses to the presence of competition and that (c) individuals competing against females will fare worse than individuals competing against males, due to the greater resource requirements of females.

## 2 METHODS

### 2.1 Study species

We used beetles obtained from a stock population kept in the University of Western Australia since 2005 on black‐eyed beans (*Vigna unguiculata*). This stock was maintained in our laboratory (at the Australian National University) in cultures of >500 beetles at 28 ± 1°C with a 14:10‐hr light:dark cycle for three generations prior to our experiment.

### 2.2 Experimental design

To test how the presence of a larval competitor and how the sex of that competitor affect adult male and female fitness traits, we conducted an experiment with a 2 × 3 factorial design, varying the sex of the focal individual (male, female) and the type of competition they experienced (none, male, female).

**FIGURE 1:**
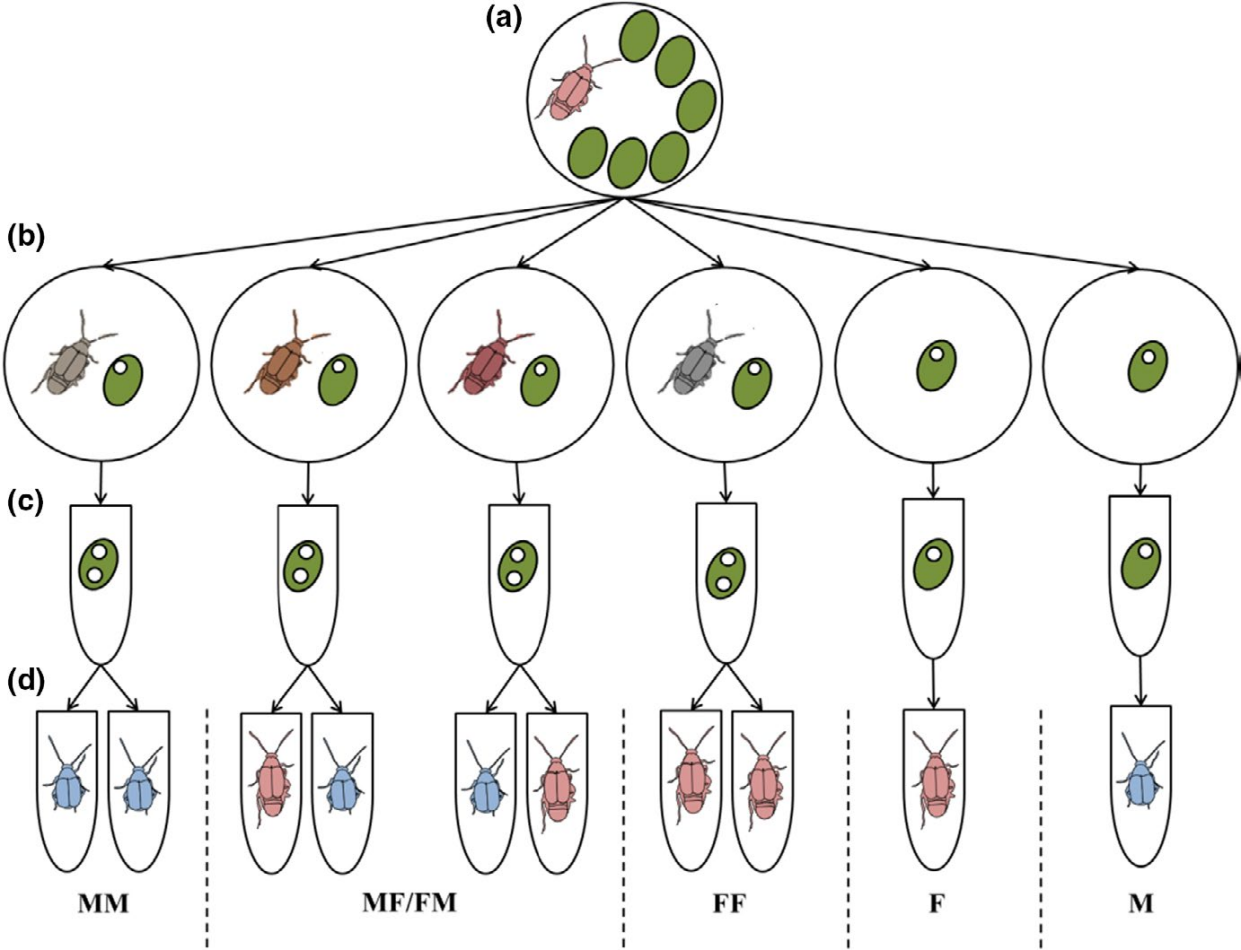
Experimental design. Four two‐egg beans and two one‐egg beans were produced per mother. The green ovals represent mung beans, and the small white circles on the mung beans represent seed beetle eggs. The sex of the seed beetles is represented by their color (pink for female and blue for male), while the treatments are represented by a mono‐ or digraph at the bottom of the diagram. In panel (b), each female has a slightly different color to differentiate between them and the original mother in panel (a)

To set up replicates of our experimental treatments, females were selected randomly from our stock population and placed in a petri dish with an ad libitum quantity of mung beans (*Vigna radiata*). We used mung beans in our experiment because these beans have enough food resources to support two larvae, but they are sufficiently limiting for effects of competition to be obvious when two larvae are present (Iglesias‐Carrasco et al., 2018; Vamosi, 2005). Females were left for half an hour to oviposit on the beans (Figure 1a). Six beans with eggs were then selected from each petri dish, and any beans with more than one egg had excess eggs scraped off. The location of the egg was marked, and each bean was placed individually in a new petri dish. To create the competitor treatments, four new females were randomly selected from our stock population and placed individually with four of the individual beans with eggs produced by each initial female. These females were observed until they laid an egg on the bean (Figure 1b). We set up a total of ~300 beans with eggs for the experiment. Since we could not tell the sex of individuals at the time of setting up the replicates, we set up 200 trials with competition and 100 without competition, on the expectation that, assuming an equal sex ratio, this would result in around 50 replicates per treatment.

Once eggs had been laid, all beans were placed into individually labeled Eppendorf tubes with a hole in the lid (Figure 1c). Tubes were kept in a temperature‐controlled room at 28°C throughout larval development. After 15 days, each Eppendorf tube was checked daily for emerged adults. At emergence, adults were transferred to their own Eppendorf tube and given a unique identification number. In the case of two‐egg beans, unless both beetles emerged on the same day, the Eppendorf tube was returned to the temperature‐controlled room until the second beetle emerged (between 1 and 5 days later), at which point the second beetle was also transferred to its own Eppendorf tube with a unique label. On the day of emergence, we identified the sex of each beetle, recorded the body weight of all beetles, measured male mating behavior and ejaculate mass, and mated females for subsequent fecundity assays (see below for details).

Once beetles emerged from their beans as adults, we were able to determine the sample sizes in each of our 6 treatments. These were as follows: male versus male (MM), *N* = 98; male versus female (M/F), *N* = 59; female versus male (FM), *N* = 59; female versus female (FF), *N* = 60; female only (F), *N* = 43; and male only (M), *N* = 45. For beans in the competition treatments, we recorded fitness traits for both beetles that emerged, irrespective of which one emerged first. That is, both beetles emerging from the beans were treated as focal individuals. This means that our sample sizes for the MF and FM treatments are identical and that our sample sizes of MM and FF treatments came from half as many beans. We fitted “bean identity” as a random effect in our mixed‐model analyses (see below) to account for the repeated measures on each bean.

### 2.3 Measurement of adult fitness traits

#### 2.3.1 Emergence weight and development time

We measured weight and development time for all emerging beetles. Development time was measured, in days, as the difference in days between the date on which the eggs were laid and the date of emergence. On the day beetles emerged, they were briefly cooled on crushed ice to slow their movement and weighed using an electronic scale with an accuracy of 0.001 mg.

#### 2.3.2 Sex‐specific fitness traits

We measured sex‐specific fitness traits for a subsample of the emerged beetles due to time constraints related to all individuals emerging in a narrow window of time (sample size: MM: 56; MF: 26; FM: 38; FF: 39; F: 36; M: 25).

Male fitness traits were copulation duration, ejaculate mass, and lifespan. On the day that a male emerged, after he had his emergence weight recorded, he was used in a mating trial in which we recorded his copulation duration. To conduct a mating trial, a male was placed in an Eppendorf tube with a randomly selected stock female. Once the male was added to the tube, we noted the time at which copulation began. We then continued to observe the beetles until they separated. The time between when mating began and when the beetles separated was used as the “copulation duration.” After mating, we reweighed the male to estimate the ejaculate mass loss (i.e., “emergence weight” minus “post‐copulation weight”; see “Emergence weight” for details on measurement collection). Ejaculate mass has previously been shown to be strongly associated with male reproductive success in this species (Katsuki, Toquenaga, & Miyatake, 2013). After the mating trial, we returned males to individual Eppendorf tubes at 28°C. Male survival was monitored daily, and lifespan was recorded as the number of days a male survived after the day of eclosion (emergence from the bean).

Female fitness traits were lifetime fecundity, lifespan, and their offsprings’ egg–adult mortality. After being weighed on their day of emergence (*see above*), each female was individually placed in a container (12 cm diameter × 4 cm) with 21 g of mung beans (i.e., ~300 mung beans) and two randomly selected stock males. Containers were then left in a temperature‐controlled room at 28°C so that they could mate and oviposit. After 2 days, the males were replaced by two new randomly selected stock males in order to standardize female exposure to males (Rönn, Katvala, & Arnqvist, 2011). Females were allowed to lay eggs in their container until death, and female lifetime fecundity was measured by counting all eggs laid by each female. Once eggs had been counted, they were returned to the temperature‐controlled room for four weeks until all offspring had emerged from the beans. At this point, the vials were transferred to a freezer (for at least 48 hr) to euthanize emerged offspring. Emerged offspring were then counted to quantify the proportion of each female's offspring that survived to adulthood (hereafter “egg–adult survival”).

During egg laying, females were checked daily for survival. On her death, the number of days she had survived after eclosion was recorded.

### 2.4 Data analysis

To determine the effects of competition and competitor sex on male and female fitness traits, we modeled our data using a series of mixed models fitted in ASReml‐R (Butler, Cullis, Gilmour, Gogel, & Thompson, 2017). We fitted separate models for seven of the traits that we measured: development time, weight at emergence, male and female lifespan, male ejaculate mass, male copulation duration, and female lifetime fecundity. In models of traits measured at emergence (development time and weight at emergence), we included the sex of the focal beetle (male or female), the competition treatment (present or absent), and, when a competitor was present, the sex of the competitor beetle (male or female) as fixed effects in our model. We also included interactions between focal sex and competition treatment, as well as (when a competitor was present) focal sex and competitor sex. The bean from which the beetle was reared was included as a random effect in all models. Maternal ID could not be included as a random effect in our analysis, because it is impossible to know which mother the emerging beetles were related to. Note that we were able to fit competitor sex in the models because ASReml allows fitting of variables conditional on a given level of a factor, using the code at (treatment, “competition”), that is, fitting competitor sex only for focal individuals with a competitor and not for those raised alone. For each analysis, we ran two models: a “full” model containing the interactions between focal sex and competition/no competition as well as between focal sex and competitor sex, and a “main effects” model containing only main effects, so we could interpret these in the absence of any potential interactions.

For the analysis of adult fitness traits (males: ejaculate mass, copulation duration, and lifespan; females: lifetime fecundity, and lifespan), models were set up in the same way, except that—because the traits were sex‐specific—we did not include sex of the focal beetle nor its interactions as fixed effects. Lifespan was analyzed separately for males and females because any sex‐specific difference might be related to different experiences as adults. All model residuals were checked to confirm that they met the assumption of normality.

For egg–adult survival we were unable to use ASReml to conduct models with conditional factors as we did for other traits, because the method does not allow specification of binomial error structure. Instead, we used a two‐step approach using generalized linear mixed models in the package lme4. In the first step, we tested the effect of competition. For this analysis, we combined replicates from the male and female competitor treatments into one “competition” treatment group and compared it to the “no competition” treatment. In the second step, we tested whether the effect of competition differed depending on the sex of the competitor. For this analysis, we excluded data from the “no competition” treatments and compared the effects of male and female competitors. We then fitted a GLMM with a binomial error distribution. As above, to account for the fact that more than one individual could have come from the same bean, we included “bean ID” as a random effect.

For all adult fitness traits, we modeled the data both with and without the focal individual's body weight as a covariate. This allowed us to determine whether any effects of competition on male and female fitness traits are driven entirely by the effects of competition on the focal individual's body size, or whether competition also affects these traits in other ways. Full model outputs including parameter estimates and test statistics for these analyses are given in the Appendix (Tables A1, A2, A3, A4, A5, A6, A7, A8).

We also ran all models with competitor weight as a covariate, to further explore how this variable influenced our results. We do not present full model outputs for these analyses, but rather highlight how including this variable alters the key factors of interest (Table A9).

## 3 RESULTS

### 3.1 Emergence weight

**FIGURE 2:**
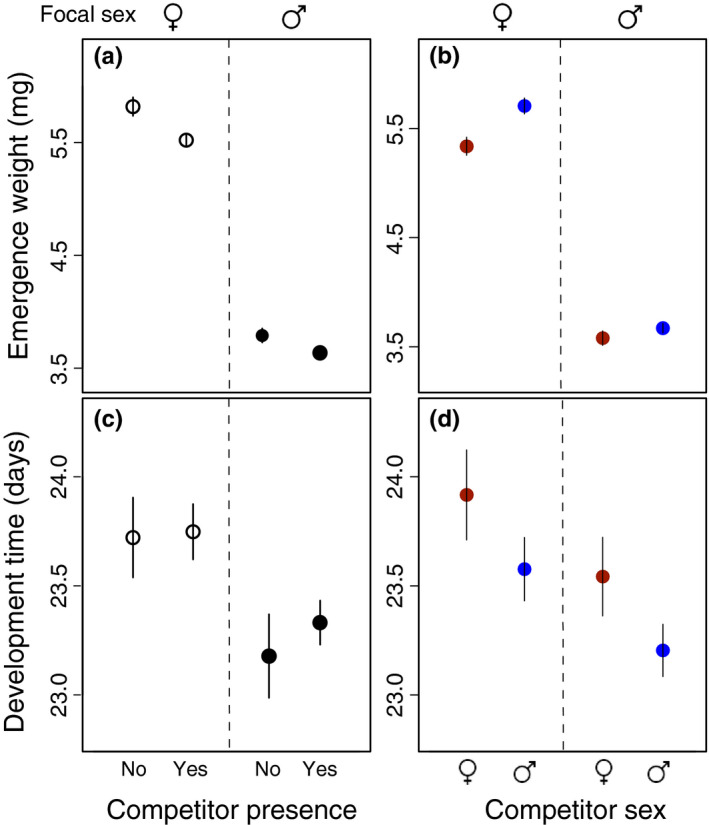
Effect of competitor presence and competitor sex (female = red; male = blue) in (a) and (b) weight at emergence, and (c) and (d) larval development time. Mean ± *SE*

Both males and females emerged lighter in the presence of a competitor than when they were raised on their own (*p* = .001, Figure 2a, Table A1). Although females emerged heavier than males (*p* < .001, Figure 2a, Table A1), there was no indication of a sex‐specific response to competition (focal sex × competition: *p* = .359). However, there was a sex‐specific response to competitor sex (focal sex × competitor sex: *p* = .031, Figure 2b): Females emerged at a lower weight when competing with another female as compared to when competing with a male (Tukey's post hoc test: *p* < .001). In contrast, male emergence weight was not significantly affected by competitor sex (post hoc Tukey's test: *p* = .720). Considering the model which also included the competitor's weight, beetles emerging from beans with larger competitors were significantly smaller; however, including competitor weight did not alter the effect of competitor sex nor the interaction between focal sex and competitor sex (Table A9).

### 3.2 Development time

Females took longer to develop than males whether a competitor was present or not (*p* = .002, Figure 2c, Table A2), but competition did not have a significant effect on the overall development time of males and females (*p* = .619; Figure 2c). Further, while there was no significant sex‐specific response to the sex of a competitor (focal sex × competitor sex: *p* = .996; Table A2), the development time of both sexes was longer when there was a female competitor present, compared to when there was a male competitor present (*p* = .017, Figure 2d). Competitor weight at emergence did not affect the development time of focal beetles, nor did it alter the effects seen for competitor sex or the focal sex‐by‐competitor sex interaction (Table A9).

### 3.3 Adult fitness traits

**FIGURE 3:**
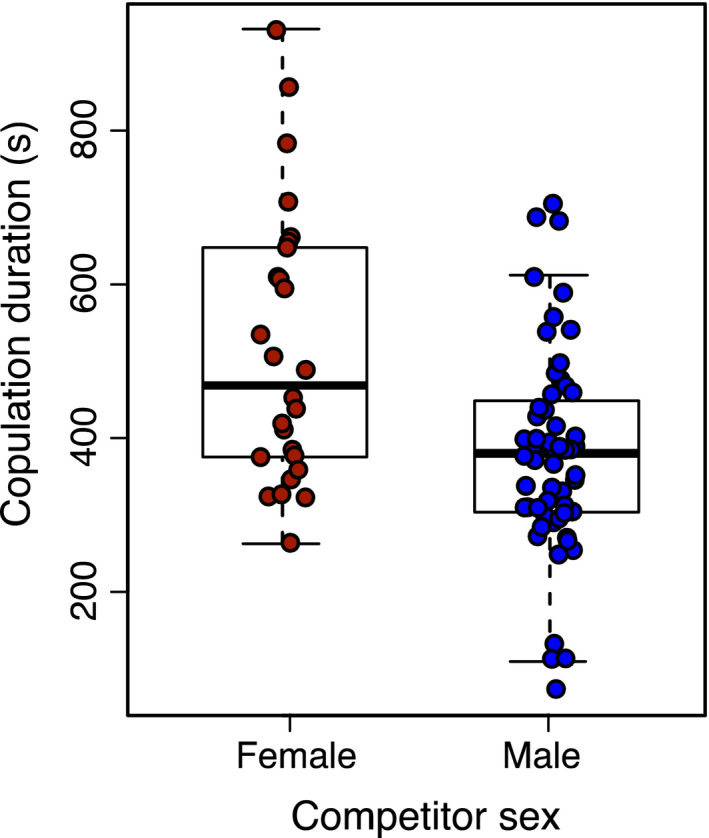
Effect of competitor sex (red = female; blue = male) on male copulation duration (s). See Figure A1 for a visual comparison with the no‐competitor treatment

In males, the presence of a competitor had no significant effects on either ejaculate mass, copulation duration, or lifespan (all *p*‐values > .10, Tables A3, A4, A5). Including emergence weight as a covariate did not alter this result, although we did also find a strong positive effect of emergence weight on both male ejaculate mass (*p* < .001, Table A3) and lifespan (*p* < .001, Table A5). When males had competed with another male during development, their copulation duration as adults was significantly shorter compared to when they had competed with a female (*p* < .001, Table A4, Figure 3). No other male fitness measures were affected by competitor sex (all *p*‐values > .471), and including the focal males emergence weight in models considering the effects of competitor sex on male fitness traits did not alter our results. Finally, as an exploratory analysis, we looked at whether there was a correlation between ejaculate mass and copulation duration. We found that, although nonsignificant, there was a weak positive association between these two variables (Pearson correlation, *r* = .21, *p* = .060).

Competitor weight at emergence had no effect on the focal male lifespan or male ejaculate mass, nor did it alter the interpretation of the competitor sex effect noted above (Table A9). In contrast, competitor weight at emergence was strongly correlated with male copulation duration, with males reared with larger competitors having longer copulation durations. However, when included together in a model with competitor sex, competitor sex remained significant (or marginally so) and the effect of competitor sex was stronger than that of competitor weight (Table A9).

For females, the presence of a competitor had no significant effect on lifetime fecundity, lifespan, or offspring egg–adult survival (all *p*‐values > .159, Tables A6, A7, A8, A9). Including the focal female's emergence weight as a covariate in these analyses did not alter our results, although we did find that larger females had higher fecundity (*p* < .001, Table A6), longer lifespans (*p* = .002, Table A7), and offspring with higher egg–adult survival (*p* = .001, Table A8). Competitor sex had no effect on female lifetime fecundity or lifespan, either before (all *p*‐values > .077) or after (all *p*‐values > .114) controlling for female emergence weight in our models.

Females competing with another female had lower egg–adult survival than those competing with a male (*p* = .030). However, this effect of competitor sex disappeared when the focal female's emergence weight was included as a covariate in our analysis (Table A8), suggesting that the effect of competitor sex is driven by the negative effect that competing with another female has on body mass.

Including competitor weight in our models did not influence the effects of competitor sex on female lifespan, but it did alter conclusions for both the lifetime number of eggs laid by females and the survival of these eggs to adulthood (Table A9). When including competitor weight in models that did not include female emergence weight as a covariate, we found: (a) when considering the number of eggs laid by females throughout their life, neither competitor sex nor competitor weight have a significant effect when included on their own, but when modeled together, both effects become significant (Table A9). This suggests that competitor sex and competitor weight have counteracting effects; (b) when considering survival of eggs laid by females reared with either males or females, both competitor weight and competitor sex have a significant effect on egg‐to‐adult survival when included on their own. However, when modeled together, the effect of competitor sex disappears and competitor weight remains, suggesting that much of the variation in egg‐to‐adult survival that is explained by competitor sex can be attributed to variation in the competitor's weight (Table A9). When including competitor weight in models that also controlled for focal female emergence weight, we see similar patterns, but they are diluted.

## 4 DISCUSSION

We examined how the sex of conspecific competitors interacts with the sex of focal individuals to drive sex‐specific responses in a range of fitness traits in the seed beetle *C. maculatus*. We found that both the sex of the focal individual and that of the competitor, as well as the interaction between the two, can affect the outcome of competition—and hence the fitness of individuals. We suggest that male and female responses to competition in seed beetles are primarily driven by competition over food resources rather than predicted future reproductive opportunities: Our results are largely consistent with the idea that being reared with resource‐hungry females has greater impacts on fitness‐related traits than being reared with males. This is not completely surprising since a beetle has a 50:50 chance of being in a bean with one sex or the other, so finding themselves with one or the other is unlikely to provide meaningful information about population sex ratio at emergence.

Both males and females took longer to develop when competing with a female than when competing with a male. This suggests there is asymmetry in the competitive abilities of males and females in *C. maculatus* and supports previous studies which show that individuals of the larger sex exert stronger competition by acquiring resources at the expense of the other (Bedhomme et al., 2003; Oddie, 2000). In addition, we found that females competing with other females were smaller at emergence than females competing with males. This was not the case for males. This finding indicates that seed beetles respond in a sex‐specific manner to competitor sex, and supports previous studies which demonstrate that, under resource limitation, the larger sex is at a disadvantage due to the costs of producing a large body (Benito & González‐Solís, 2007; Wikelski & Thom, 2000). Our results regarding the effects of competition on development time and emergence weight highlight the importance of exploring how the sex of both focal individuals and their competitors influences the outcome of the competition, since the effect of the competitor sex might not always mirror the effect of the focal sex.

We found no effect of competition or competitor sex on male or female lifespan, whether or not emergence weight was included as covariate in the model. However, lifespan was positively correlated with emergence weight in both sexes, as has been previously found in seed beetles (Iglesias‐Carrasco et al., 2018). The lack of an effect of competition on lifespan when emergence weight is not included in the model is somewhat surprising given the effects of competition on emergence weight and the relationship between emergence weight and lifespan. Although we cannot completely discard the possibility that this lack of effect is the result of low statistical power, our results suggest either that beetles somehow compensate for the effects that competition has on lifespan or more likely that the effects of competition on weight are not sufficiently strong to translate to lifespan.

Competition had no detectable effects on a female's lifetime fecundity or the subsequent egg–adult survival of her offspring. However, there was an effect of competitor sex: Females that had competed with another female as larvae had offspring with lower egg–adult survival than those that had competed with a male. Adult seed beetles are facultatively aphagous (Messina & Slade, 1997). As such, conditions experienced as larvae are expected to be important in determining the allocation of resources to traits later in life. Females that competed with other females are likely to have experienced stronger food limitation than those reared with males. Previous studies of a related species have shown that unfed or food‐stressed females alter the partitioning of resources to each egg, laying smaller eggs (Yanagi & Miyatake, 2002), and smaller eggs have been shown previously to have lower offspring survival (Fox, 1993). When we controlled for female emergence weight in our analysis, we found that larger females had offspring that were more likely to survive to adulthood and that the effect of competitor sex became nonsignificant. Likewise, when we controlled for competitor weight in our analysis, females reared with larger beetles had offspring with lower egg–adult survival and the effect of competitor sex became nonsignificant. This suggests that the effect of female competitors on egg–adult survival is mediated through reduced body size, which is most likely caused by competition over food resources.

As in many previous studies of seed beetles (Chou et al., 2016; Iglesias‐Carrasco et al., 2018), larger females had greater lifetime fecundity. However, our results contrast with a previous study in seed beetles where competition directly affected fecundity, but had no effect on emergence weight (Vamosi, 2005). Our study shows that competition affects body size and that body size affects fecundity, but fails to find either direct or indirect (via body size) effects of competition on fecundity. This could indicate that, as for survival, the effects of competition on adult phenotypes are not enough to substantially affect fecundity. Although, when we look at the effects of competitor sex in combination with those of competitor weight—in an attempt to tease apart the effects of competition over resources from the effects of social environment—we see that while neither competitor sex nor competitor weight affect female lifetime fecundity when modeled alone, they are both significant when modeled together: Females reared with males subsequently produce more eggs, while females reared with large competitors also produce more eggs. This counteracting effect might seem counterintuitive at first, but could arise if differences in competitor size beyond those related to sex result from the quality or quantity of resources available. Such results highlight the difficulty in teasing apart effects of resource availability from other effects in competition studies.

**FIGURE A1:**
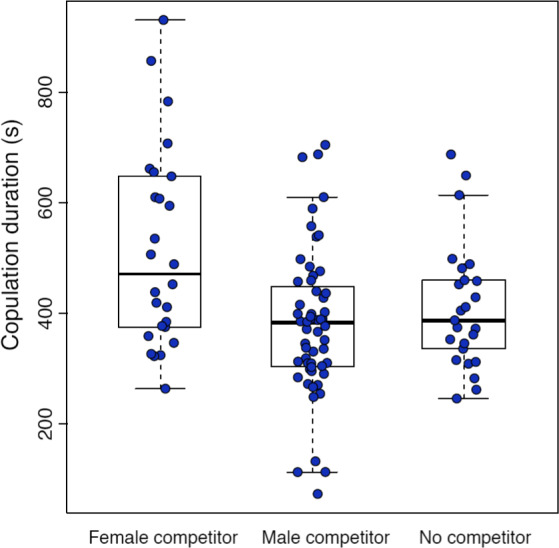
Effect of competitor sex (female and male), and no competitor on male copulation duration (s)

The presence of a competitor, independent of its sex, did not affect male ejaculate mass or copulation duration. We also found no effect of competitor sex on ejaculate mass. *C. maculatus* males can reduce female remating and hence sperm competition by transferring large ejaculates that provide direct benefits to females (Yamane, Goenaga, Rönn, & Arnqvist, 2015; Yamane, Miyatake, & Kimura, 2008), by transferring substances that increase refractory periods in females (Hotzy, Polak, Rönn, & Arnqvist, 2012), or by performing longer copulations that increase the costs associated with mating (Crudgington & Siva‐Jothy, 2000; Edvardsson & Tregenza, 2005). Therefore, we expected increased investment in ejaculate mass and copulation duration for males competing with other males due to a perceived greater risk of reproductive competition (Bretman et al., 2011). In contrast, we found that males had longer copulations when they had competed with a female (Figure A1). If the social environment is driving this pattern, one potential explanation is that males reduce their investment in individual copulations to maximize the number of females they mate with when competitors are around. However, this seems unlikely given that it was males reared with females (and not those reared with males) that changed their behavior relative to the no‐competitor treatment. Another possible explanation may be that this result is driven by competition for resources. Under such a scenario, males that competed with females might be in worse condition than those competing with males due to the greater resource acquisition abilities of females. If males in poor condition transfer ejaculates more slowly to females, then this could lead to increased copulation durations to ensure a minimum threshold of sperm transfer. However, when we included the weight of competitors in our analysis, the effect of competitor sex remained significant (or marginally nonsignificant, depending on whether we also controlled for the weight of the focal male), suggesting that access to resources may not be the only factor driving this result. Either way, the lack of increased investment in ejaculates and copulation duration when competing with another male indicates that if changes in mating behavior result from differences in the social environment, it is not in the direction we predicted, and future studies would benefit from looking at male mating behavior in a more realistic context (i.e., in the presence versus absence of competitors) and over a longer time frame. This approach has recently been taken in Eastern mosquitofish (Spagopoulou, Vega‐trejo, Head, & Jennions, 2020).

## 5 CONCLUSIONS

We show that the sex of the competitor plays an important role in driving sex‐specific responses to competition and that the larger sex, in this case the female, exerts stronger competitive pressure than the smaller sex. We also found that females responded more strongly to the presence of competitor females than males did. Our results indicate that, in addition to sex‐specific responses to resource variability, the sex of the competitor is also an important determinant of fitness traits. The effects observed in our study might be the consequence of a combination between asymmetry in the competitive abilities of males and females, and sex‐specific differences in food requirements. It is less likely, however, that the sex‐specific responses to competitor sex are driven by changes in the motivation of competing individuals due to anticipated future intrasexual competition over reproduction. Our study highlights the importance of considering the sex of competitors in studies of phenotypic responses to competition.

## CONFLICT OF INTEREST

The authors declare no competing interests.

## AUTHOR CONTRIBUTIONS

**Maider Iglesias‐Carrasco:** Formal analysis (equal); writing – original draft (lead). **Samuel Brookes:** Conceptualization (equal); investigation (lead); writing – review and editing (equal). **Loeske E. B. Kruuk:** Conceptualization (equal); formal analysis (equal); writing – review and editing (equal). **Megan L. Head:** Conceptualization (equal); formal analysis (equal); supervision (lead); writing – review and editing (equal).

## Data Availability

Data are available from DRYAD https://doi.org/10.5061/dryad.573n5tb52.

## APPENDIX 1

**TABLE A1:** Effects of competition (present/absent) and competitor sex (male/female) on **weight at emergence (mg)** of both focal males and females

Terms	Estimate	*SE*	*df*	*F*	*p*
Full model					
Fixed effects					
Intercept	5.337	0.067	1,206.9	16,470	<.001
Focal sex (male)	−1.758	0.095	1,354.9	1,271	<.001
Competition (no)	0.481	0.103	1,328.7	12.350	<.001
Competition × Focal sex	0.270	0.145	1,358.0	**0.844**	**.359**
Within competition treatment effects					
Competitor sex (male)	0.370	0.095	1,353.3	11.820	**<.001**
Competitor sex × Focal sex	−0.279	0.128	1,158.1	4.752	**.031**
**Random effects**	**Variance**	***SE***			
Bean ID	0.007	0.022			
**Residual**	**0.256**	**0.029**			
Main effects model					
Fixed effects					
Intercept	5.428	0.055	1,209.3	16,140	<.001
Focal sex (male)	−1.941	0.055	1,358.1	1,260	**<.001**
Competition (no)	0.345	0.073	1,329.4	12.17	**<.001**
Within competition treatment effects					
Competitor sex (male)	0.218	0.063	1,358.8	12.02	**<.001**
**Random effects**	**Variance**	***SE***			
Bean ID	0.010	0.022			
Residual	0.255	0.029			

Note

‘Full models’ provide parameter estimates and significance values for interpreting the interaction effect (main effects in these models (grey text) are shown for transparency, but should not be interpreted). ‘Main effects’‐only models do not fit interactions, and provide parameter estimates and significance values for interpreting the main effects. Results are from mixed models fitted in ASReml‐R. Factors related to characteristics of competitors were fit as conditional effects. *F* statistics are conditional and taken from Wald's test. *p*‐values < .05 are shown in bold. Shaded rows show the effects of primary interest.

**TABLE A2:** Effects of competition (present/absent) and competitor sex (male/female) on **development time** (days) of both focal males and females

Terms	Estimate	*SE*	*df*	*F*	*p*
Full model					
Fixed effects					
Intercept	23.917	0.209	1,219.3	61,410	<.001
Focal sex (male)	−0.374	0.268	1,302.7	9.516	.002
Competition (no)	−0.196	0.287	1,256.5	0.246	.621
Competition × Focal sex	−0.169	0.385	1,299.0	0.290	.591
Within competition treatment effects					
Competitor sex (male)	−0.340	0.268	1,316.1	4.912	**.027**
Competitor sex × Focal sex	0.002	0.398	1,186.8	0.000	.996
**Random effects**	**Variance**	***SE***			
Bean ID	0.932	0.146			
Residual	0.747	0.089			
Main effects model					
Fixed effects					
Intercept	23.949	0.166	1,221.1	65,290	<.001
Focal sex (male)	−0.413	0.133	1,314.0	9.575	**.002**
Competition (no)	−0.295	0.190	1,258.7	0.248	.619
Within competition treatment effects					
Competitor sex (male)	−0.357	0.149	1,350.3	5.769	**.017**
**Random effects**	**Variance**	***SE***			
Bean ID	0.922	0.144			
Residual	0.747	0.089			

Note

‘Full models’ provide parameter estimates and significance values for interpreting the interaction effect (main effects in these models (grey text) are shown for transparency, but should not be interpreted). ‘Main effects’‐only models do not fit interactions, and provide parameter estimates and significance values for interpreting the main effects. Results are from mixed models fitted in ASReml‐R. Factors related to characteristics of competitors were fit as conditional effects. *F* statistics are conditional and taken from Wald's test. *p*‐values < .05 are shown in bold. Shaded rows show the effects of primary interest.

**TABLE A3:** Effects of competition (present/absent) and competitor sex (male/female) on **male ejaculate mass (mg)**

Terms	Estimate	*SE*	*df*	*F*	*p*
Not controlling for male weight					
Fixed effects					
Intercept	0.204	0.013	1,73.1	554.9	<.001
Competition (no)	0.016	0.018	1,95.4	2.474	.119
Within competition treatment effects					
Competitor sex (male)	−0.011	0.016	1,83.1	0.526	.471
**Random effects**	**Variance**	***SE***			
Bean ID	0.001	0.001			
Residual	0.003	0.001			
Controlling for male weight					
Fixed effects					
Intercept	−0.001	0.057	1,73.0	0.007	.933
Competition (no)	0.015	0.015	1,95.2	2.104	.150
Male weight	0.055	0.015	1,99.0	13.570	**<.001**
Within competition treatment effects					
Competitor sex (male)	−0.891	1.476	1,83.2	0.365	.547
**Random effects**	**Variance**	***SE***			
Bean ID	0.001	0.001			
Residual	0.003	0.001			

Note

We ran models twice – first without including focal male weight (“Not controlling for male weight) and then with focal male weight (“Controlling for male weight”). Results are from mixed models fitted in ASReml‐R. Factors related to characteristics of competitors were fit as conditional effects. *F* statistics are conditional and taken from Wald's test. *p*‐values < .05 are shown in bold. Shaded rows show the effects of primary interest.

**TABLE A4:** Effects of competition (present/absent) and competitor sex (male/female) on **male copulation duration (s)**

Terms	estimate	*SE*	*df*	*F*	*p*
Not controlling for male weight					
Fixed effects					
Intercept	514.830	27.751	1,104	549.100	<.001
Competition (no)	−103.066	39.636	1,104	0.121	.729
Within competition treatment effects					
Competitor sex (male)	−134.475	33.581	1,104	16.040	**<.001**
**Random effects**	**Variance**	***SE***			
Bean ID	0.008	0.001			
Residual	20,022	2,776			
Controlling for male weight					
Fixed effects					
Intercept	692.754	134.124	1,103	23.890	<.001
Competition (no)	−101.839	39.488	1,103	0.077	.782
Male weight	−47.267	34.866	1,103	1.838	.178
Within competition treatment effects					
Competitor sex (male)	−136.569	33.482	1,103	16.640	**<.001**
**Random effects**	**Variance**	***SE***			
Bean ID	0.009	0.001			
Residual	19,862	2,767			

Note

We ran models twice – first without including focal male weight (“Not controlling for male weight) and then with focal male weight (“Controlling for male weight”). Results are from mixed models fitted in ASReml‐R. Factors related to characteristics of competitors were fit as conditional effects. *F* statistics are conditional and taken from Wald's test. *p*‐values < .05 are shown in bold. Shaded rows show the effects of primary interest.

**TABLE A5:** Effects of competition (present/absent) and competitor sex (male/female) on **male lifespan** (days)

Terms	Estimate	*SE*	*df*	*F*	*p*
Not controlling for male weight					
Fixed effects					
Intercept	16.423	0.894	1,104	630.200	<.001
Competition (no)	−0.823	1.277	1,104	1.624	.497
Within competition treatment effects					
Competitor sex (male)	0.738	1.081	1,104	0.465	.497
**Random effects**	**Variance**	***SE***			
Bean ID	0.000	0.000			
Residual	20.768	2.880			
Controlling for male weight					
Fixed effects					
Intercept	0.075	4.035	1,103	0.006	.938
Competition (no)	−0.936	1.188	1,103	2.612	.109
Male weight	4.343	1.049	1,103	17.140	**<.001**
Within competition treatment effects					
Competitor sex (male)	0.930	1.007	1,103	0.853	.358
**Random effects**	**Variance**	***SE***			
Bean ID	0.000	0.000			
Residual	17.977	2.505			

Note

We ran models twice – first without including focal male weight (“Not controlling for male weight) and then with focal male weight (“Controlling for male weight”). Results are from mixed models fitted in ASReml‐R. Factors related to characteristics of competitors were fit as conditional effects. *F* statistics are conditional and taken from Wald's test. *p*‐values < .05 are shown in bold. Shaded rows show the effects of primary interest.

**TABLE A6:** Effects of competition (present/absent) and competitor sex (male/female) on **female lifetime number of eggs**

Terms	Estimate	*SE*	*df*	*F*	*p*
Not controlling for female weight					
Fixed effects					
Intercept	92.362	3.214	1,88.5	1949	<.001
Competition (no)	4.832	4.328	1,97.6	0.554	.458
Within competition treatment effects					
Competitor sex (male)	3.822	4.277	1,68.3	0.799	.375
**Random effects**	**Variance**	***SE***			
Bean ID	103.766	62.683			
Residual	198.830	59.302			
Controlling for female weight					
Fixed effects					
Intercept	−5.700	12.564	1,109	0.121	.729
Competition (no)	−2.358	3.351	1,109	0.124	.725
Female weight	18.193	2.299	1,109	62.600	**<.001**
Within competition treatment effects					
Competitor sex (male)	−2.529	3.280	1,109	0.595	.442
**Random effects**	**Variance**	***SE***			
Bean ID	5.846E−04	7.919E−06			
Residual	193.760	26.246			

Note

We ran models twice – first without including focal female weight (“Not controlling for female weight) and then with focal female weight (“Controlling for female weight”). Results are from mixed models fitted in ASReml‐R. Factors related to characteristics of competitors were fit as conditional effects. *F* statistics are conditional and taken from Wald's test. *p*‐values < .05 are shown in bold. Shaded rows show the effects of primary interest.

**TABLE A7:** Effects of competition (present/absent) and competitor sex (male/female) on **female lifespan** (days)

Terms	Estimate	*SE*	*df*	*F*	*p*
Not controlling for female weight					
Fixed effects					
Intercept	6.704	0.299	1,56.1	1,372	<.001
Competition (no)	0.524	0.387	1,58.7	2.030	.159
Within competition treatment effects					
Competitor sex (male)	0.143	0.375	1,45.8	0.145	.705
**Random effects**	**Variance**	***SE***			
Bean ID	0.805	0.304			
Residual	0.511	0.216			
Controlling for female weight					
Fixed effects					
Intercept	2.988	1.223	1,55.8	5.827	.019
Competition (no)	0.167	0.397	1,58.7	0.881	.352
Female weight	0.704	0.225	1,56.9	9.813	**.002**
Within competition treatment effects					
Competitor sex (male)	−0.163	0.382	1,51.4	0.183	.670
**Random effects**	**Variance**	***SE***			
Bean ID	0.884	0.261			
Residual	0.342	0.148			

Note

We ran models twice – first without including focal female weight (“Not controlling for female weight) and then with focal female weight (“Controlling for female weight”). Results are from mixed models fitted in ASReml‐R. Factors related to characteristics of competitors were fit as conditional effects. *F* statistics are conditional and taken from Wald's test. *p*‐values < .05 are shown in bold. Shaded rows show the effects of primary interest.

**TABLE A8:** Effects of competition (present/absent) and competitor sex (male/female) on female egg−adult survival

Terms	Estimate	*SE*	*z*	*p*
Effect of competition				
Not controlling for female weight				
Fixed effects				
Intercept	1.799	0.069	26.041	<.001
Competition (no)	0.078	0.114	0.687	.492
**Random effects**	**Variance**	***SD***		
Bean ID	0.199	0.446		
Controlling for female weight				
Fixed effects				
Intercept	0.462	0.422	1.095	.273
Competition (no)	0.034	0.116	0.291	.771
Female weight	**0.238**	**0.075**	**3.199**	**.001**
**Random effects**	**Variance**	***SD***		
Bean ID	0.208	0.455		
Effect of competitor sex				
Not controlling for female weight				
Fixed effects				
Intercept	1.626	0.093	17.424	<.001
Competitor sex (male)	**0.261**	**0.120**	**2.166**	**.030**
**Random effects**	**Variance**	***SD***		
Bean ID	0.129	0.359		
Controlling for female weight				
Fixed effects				
Intercept	0.565	0.459	1.231	.218
Competitor sex (male)	0.189	0.132	1.435	.151
Female weight	**0.198**	**0.084**	**2.361**	**.018**
**Random effects**	**Variance**	***SD***		
Bean ID	0.153	0.391		

Note

We ran each analysis twice – first without including focal female weight (“Not controlling for female weight) and then with focal female weight (“Controlling for female weight”). Results are generalised linear mixed models with a binomial error distribution. *p*‐values < .05 are shown in bold. Shaded rows show the effects of primary interest.

**TABLE A9:** Comparison of competitor effects on each trait across models including/excluding competitor sex and competitor weight

Trait	Effect	Original model (competitor sex only)			Competitor sex and competitor weight			Competitor weight only		
		Estimate	*SE*	*p*	Estimate	*SE*	*p*	Estimate	*SE*	*p*
Weight at emergence	Focal sex × Competitor sex	−0.279	0.128	.031	−0.274	0.128	.033			
	Competitor sex	0.218	0.063	<.001	0.300	0.129	.021			
	Competitor weight				0.042	0.059	.474	−0.077	0.029	.008
Development time	Focal sex × Competitor sex	0.002	0.398	.996	−0.032	0.397	.935			
	Competitor sex	−0.357	0.149	.017	−0.583	0.284	.041			
	Competitor weight				−0.117	0.125	.353	0.092	0.068	.177
Male ejaculate mass (not controlling for focal male weight)	Competitor sex	−0.011	0.016	.471	−6.431	3.505	.069			
	Competitor weight				−2.682	1.586	.094	−0.038	0.712	.957
Male ejaculate mass (controlling for focal male weight)	Competitor sex	−0.891	1.476	.547	−4.021	3.402	.239			
	Competitor weight				−1.578	1.546	.310	0.068	0.672	.919
Male copulation duration (not controlling for focal male weight)	Competitor sex	−134.475	33.581	<.001	−151.007	78.924	.058			
	Competitor weight				−8.369	36.119	.817	54.107	15.635	<.001
Male copulation duration (controlling for focal male weight)	Competitor sex	−136.569	33.482	<.001	−164.927	79.178	.039			
	Competitor weight				−14.318	36.179	.693	54.015	15.614	<.001
Male lifespan (not controlling for focal male weight)	Competitor sex	0.738	1.081	.497	−1.579	2.530	.534			
	Competitor weight				−1.173	1.158	.313	−0.519	0.493	.295
Male lifespan (controlling for focal male weight)	Competitor sex	0.930	1.007	.358	−0.438	2.378	.854			
	Competitor weight				−0.691	1.087	.527	−0.509	0.459	.270
Female lifetime number of eggs (not controlling for focal female weight)	Competitor sex	3.822	4.277	.375	19.324	7.308	.009			
	Competitor weight				8.918	3.619	.015	−0.068	2.064	.974
Female lifetime number of eggs (controlling for focal female weight)	Competitor sex	−2.529	3.280	.442	9.827	5.975	.103			
	Competitor weight				7.126	2.907	.016	3.092	1.573	.052
Female lifespan (not controlling for focal female weight)	Competitor sex	0.143	0.375	.705	−0.614	0.605	.313			
	Competitor weight				−0.504	0.311	.113	−0.237	0.198	.234
Female lifespan (controlling for focal female weight)	Competitor sex	−0.163	0.382	.670	−0.581	0.576	.317			
	Competitor weight				−0.295	0.305	.337	−0.065	0.202	.749
Female egg to adult survival (not controlling for focal female weight)	Competitor sex	0.261	0.120	.030	−0.175	0.196	.372			
	Competitor weight				−0.253	0.086	.003	−0.195	0.056	<.001
Female egg to adult survival (controlling for focal female weight)	Competitor sex	0.189	0.132	.151	−0.126	0.197	.522			
	Competitor weight	0.198	0.084	.018	−0.201	0.094	.032	−0.156	0.062	.012
